# ‘CTRL’: an online, Dynamic Consent and participant engagement platform working towards solving the complexities of consent in genomic research

**DOI:** 10.1038/s41431-020-00782-w

**Published:** 2021-01-06

**Authors:** Matilda A. Haas, Harriet Teare, Megan Prictor, Gabi Ceregra, Miranda E. Vidgen, David Bunker, Jane Kaye, Tiffany Boughtwood

**Affiliations:** 1 Australian Genomics Health Alliance, Parkville, VIC Australia; 2 Murdoch Children’s Research Institute, Parkville, VIC Australia; 3 Centre for Health, Law and Emerging Technologies, Faculty of Law, University of Oxford, Oxford, UK; 4 Centre for Health, Law and Emerging Technologies, Melbourne Law School, University of Melbourne, Carlton, VIC Australia; 5 Curve Tomorrow, Melbourne, VIC Australia; 6 QIMR Berghofer Medical Research Institute, Herston, QLD Australia; 7 Queensland Genomics Health Alliance, Herston, QLD Australia

**Keywords:** Genetics research, Patient education

## Abstract

The complexities of the informed consent process for participating in research in genomic medicine are well-documented. Inspired by the potential for Dynamic Consent to increase participant choice and autonomy in decision-making, as well as the opportunities for ongoing participant engagement it affords, we wanted to trial Dynamic Consent and to do so developed our own web-based application (web app) called CTRL (control). This paper documents the design and development of CTRL, for use in the Australian Genomics study: a health services research project building evidence to inform the integration of genomic medicine into mainstream healthcare. Australian Genomics brought together a multi-disciplinary team to develop CTRL. The design and development process considered user experience; security and privacy; the application of international standards in data sharing; IT, operational and ethical issues. The CTRL tool is now being offered to participants in the study, who can use CTRL to keep personal and contact details up to date; make consent choices (including indicate preferences for return of results and future research use of biological samples, genomic and health data); follow their progress through the study; complete surveys, contact the researchers and access study news and information. While there are remaining challenges to implementing Dynamic Consent in genomic research, this study demonstrates the feasibility of building such a tool, and its ongoing use will provide evidence about the value of Dynamic Consent in large-scale genomic research programs.

## Introduction

Research in genomic medicine creates new and unique challenges for the informed consent process [1, 2]. Consent must cover the potential for unexpected findings, family implications, health information privacy and data sharing for further research. Given this complexity, genomic research information and consent forms can be very long. It can be difficult to convey all of the necessary information and ensure participant understanding during a study recruitment and consent appointment [3, 4].

Broad consent to future use of research participant data has often been used in established genomic research programs [5]. It has been a practical model for many reasons, including low administrative burden compared to the alternative of more granular and updateable consent models. However, requesting broad consent for use of each individual’s genomic data may affect research participation rates (particularly from certain groups in society) due to concerns about the privacy of personal data [6]. Some participants may risk missing out on the benefits of research opportunities if they are not willing to agree to broad future use [7].

This paper documents the development process for the web-based application (web app) CTRL (control), so that it can help others develop digital approaches to improve patient consent, communication and engagement in genomic research. An evaluation of patient experiences of using CTRL in genomic research will follow in a later, separate study.

## Dynamic Consent

Dynamic Consent [8] provides the conceptual framework to address many of the complex issues associated with consent and enhancing participant engagement in research. Dynamic Consent uses internet-based platforms to create a communication interface to support ongoing participant-led management of their involvement in research studies. It allows participants to: develop greater understanding of the research; choose from more granular consent options and change consent choices over time (including for future use of their data); indicate preferences for return of results; and engage in the research process as much as they choose, all according to their own time frames. In the context of this study, participant engagement means providing information about research to participants, where this information equips people to be able to take control over decisions. This is done by providing opportunities for: two-way communication; to make changes to their choices about participation, and to be kept informed about the study. We and others consider participant engagement imperative for gaining trust and minimising privacy concerns, which in turn may improve uptake and retention of participants when it comes to ongoing data sharing initiatives like genomic research and biobanks [9].

Dynamic Consent also provides the opportunity for people to understand how their contribution to research supports knowledge discovery for better health outcomes, an important aspect of precision healthcare and learning health systems. Precision healthcare is driven by linking individuals’ biological samples and information, including genomic and other health records. The comprehensive, unique and identifiable nature of personal information held about an individual, combined with data-sharing, open-ended research and the new challenges to broad consent models (for example, with the introduction of European General Data Protection Regulation), lend precision medicine initiatives to eConsent, and more specifically, Dynamic Consent approaches [10–12]. Dynamic Consent may facilitate data availability for precision medicine by increasing efficiencies in managing patient consent, and can be used to facilitate individual patient preferences about the degree of linkage of personal data.

Further, by its design, Dynamic Consent also offers potential advantages to research organisations and healthcare-delivery organisations engaged in research. These include: enabling better record-keeping of consent, in an electronic rather than paper-based format [13]; facilitating clearer data governance frameworks; improving retention of participants in long-term or longitudinal studies and providing the potential to unlock information collected for healthcare to be used in research.

### Application to the Australian Genomics study

The Australian Genomics study is a health services research project building evidence to inform the integration of genomic medicine into mainstream healthcare [14]. The programme is prospectively recruiting up to 5000 patients for genomic testing through 18 different rare disease and cancer clinical flagship projects. Given the intention for Australian Genomics to establish good practice for future genomics research, we wanted to trial the Dynamic Consent approach by developing our own platform called CTRL (reflecting the use of an IT-based delivery mechanism to give participants greater control over research participation). The design, development and implementation of CTRL required a multi-disciplinary effort, and gave rise to both expected and unexpected challenges. Reporting on the development process for CTRL responds to a recent call to build the evidence base for Dynamic Consent [15], and will be useful for research organisations pursuing this approach as it shifts from an aspirational best practice towards implementation into research use.

## Methods

### Project working group

In July 2017 a multi-disciplinary working group of 12 members from the Australian Genomics research network was established to deliver the project. Acknowledging the importance for research studies to incorporate consumer and community involvement (https://www.nhmrc.gov.au/guidelinesforguidelines/plan/consumer-involvement, https://www.telethonkids.org.au/globalassets/media/images/pagessections/research/help-shape-our-research/the-green-book-mar08.pdf) [16], the group included two consumer representatives and a patient advocate. Other professions represented in the working group include a clinical geneticist; genetic counsellors; bioethicists; a patient and community education and engagement professional; health technology and data management experts and investigators who originally developed the Dynamic Consent approach. The group met by teleconference bi-monthly to discuss progress and challenges, decide on priorities and make design and development decisions. Compliance with ethical guidelines, best practice guidelines in research and clinical care and relevant laws were integral to developing the content of the platform.

### Research

The working group began with a mapping review—a detailed search of published and grey literature—to find instances where Dynamic Consent had been either implemented or piloted in research programmes like genomic studies, patient registries and biobanks. We did not focus on commercially available consent products because information about such products was less publicly available. The mapping review informed the working group’s brief to the software developer, which included an outline of potential features for inclusion in a Dynamic Consent platform, design choices, feasibility and, importantly, user acceptability.

### Design and development

A contract partnership was entered with the digital health technology company Curve Tomorrow (Curve, curvetomorrow.com) to develop the Dynamic Consent tool as a web app. Curve was the successful vendor because of their strong track-record in working with the not-for-profit sector to develop digital health products. A web app was the favoured approach for this project because desktop access would likely provide the best experience for participants given the amount and complexity of content. This also meant that the standards-based application interface design would work on smartphones, tablets, laptops and desktop computers. A web app delivery model also minimises ongoing costs associated with app updates required for purpose-built Android or iOS-based applications and reduces time-frames because of potential delays for the approval processes required with Apple and Google App stores. It also supported more frequent and agile development cycles. Potential limitations of this chosen approach over a mobile app include that participants may access mobile apps more often because they offer instant access, and that we would not be able to take advantage of push notifications to prompt interaction with the app.

The working group provided Curve with an initial brief, which was informed by the mapping review. Curve used their software design process (Curve Way^TM^), which is based on user experience (UX) and Design Thinking principles: to Empathise, Define, Create, Prototype, Test, and then release the Product. To do this, Curve initially conducted 30–60 min face-to-face semi-structured interviews with six individuals representing key stakeholder groups in the project, primarily to gain a greater understanding of the problem—the complexities of consent in genomic research—and our proposed solution, Dynamic Consent. Interviews were conducted with a genetic counsellor, a clinician researcher, a patient, parent/guardian of a patient, a patient advocate and the project lead, representing stakeholders/end user groups.

Themes arising from interviews informed the next steps: creating user flows; affinity mapping and identifying key pain points; and then the ideate/create phase focussed on addressing key pain points (using the ‘How Might We’ method). Wireframes and a clickable prototype were developed. Feedback on the design was sought from the stakeholders involved in the interview process, through one-on-one testing of the prototype. The working group also reviewed the design and prototype to provide feedback. This included consideration of the ‘backend’ system, the ability to repurpose the solution and how it would allow for scale and portability across research and clinical delivery. The implications of the software design on future intellectual property and licensing were also considered. Once the design was approved, the project was officially launched by Curve, who built the CTRL database using PostgreSQL (open-source relational database management system; www.postgresql.org) and the web app using Ruby on Rails (open-source software for building web apps; https://rubyonrails.org/). A combination of automated testing (unit-level code testing; predominant end-to-end flow) and manual testing (user validation; alternative end-to-end flow; user acceptance) was done as appropriate, at each stage of development.

The written content was developed concurrently with web development. Because the Australian Genomics study had already been approved by a Human Research Ethics Committee (HREC) and had commenced recruiting participants, the information delivered in the platform had to reflect the approved paper-based Participant Information and Consent Forms. There was an intention to harmonise CTRL with the generic clinical consent form and information materials developed by Australian Genomics and being trialled for national use [15]. The written content was reviewed widely, including by a plain language writer experienced in writing genomic testing materials for patients. Additional requirements for the website included a Privacy Policy and Terms and Conditions, which were developed by the institutional legal team.

### Security considerations

Given the sensitive nature of the personal information collected in the first version of CTRL, and potentially highly sensitive information to be stored in the system with future expansion of features, the project included a strong security focus. The assessment of security-related threats was considered during the development of requirements and the design and build of the system. The developers actively engage with health information law and guidelines including but not limited to: Therapeutic Goods Association guidelines on software and cyber security, the Medical Device Quality Standards, the Open Web Application Security Project (OWASP), the Privacy Act 1988 (Cth) and the Australian Government Information Security Manual. In addition, working group members ensured guidelines including ISO27799, AS HB174 and the National eHealth Security and Access Framework were employed.

The system is designed so that although anyone can register to use the demonstration version of the site, an invitation to register for the production website is only extended to study participants during face-to-face appointments and by follow-up email. Only people who have a Study ID number are able to register. Registration and subsequent login are password-protected, and timed log-out occurs after 10 min of inactivity. Password reset instructions can be sent to a registered email address. Secure institutional servers host the CTRL database, the REDCap [17, 18] electronic data capture tool (study database) it is integrated with, and an instance of the Metabase (metabase.com) open-source data analytic tool. All participants registering to use CTRL are asked to agree to the Terms and Conditions of use, and are encouraged to review the Privacy Policy.

### Participant data—sharing and access

Dynamic Consent platforms are able to facilitate an alternative to broad, ‘all or nothing’ consent, with the opportunity to choose from more granular consent options and revisit consent over time. Australian Genomics participates in the Global Alliance for Genomics and Health (GA4GH, ga4gh.org), the international standards-setting organisation for genomic data sharing. GA4GH’s Data Use and Researcher Identity (DURI) Workstream has developed the Data Use Ontology (DUO) technical standard (https://github.com/EBISPOT/DUO), which is a series of data access codes that can be applied to research cohort datasets to describe data access and use permissions in machine-readable formats. The working group set out to develop the series of questions presented to participants in the biological sample and data sharing section of CTRL to employ the DUO codes standard (version 1.0; [19]). Our application of DUO to this project also tests how data management processes can incorporate personalised decision-making through application of DUO codes to individuals’ data, rather than whole cohorts. Application of DUO is expected to reduce ambiguity about the meaning of consent clauses and facilitate inter-jurisdictional genomic data-sharing efforts.

## Results

### Mapping review and design

The working group’s mapping review identified a range of existing Dynamic Consent tools applied in Europe and North America. We considered the objectives and features of Dynamic Consent platforms developed for the RUDY (rare UK diseases) study (RudyStudy.org) [20, 21]; the Genetic Alliance’s patient registry Platform for Engaging Everyone Responsibly (peerplatform.org) and The Michigan BioTrust for Health blood spot biobank trial [22]. From this, the working group established a short-list of potential features that the Dynamic Consent platform could incorporate (Table 1). Features were also considered in the context of their applicability to other research studies in future: we did not want to make the features too specific to our research project. The working group decided on priorities for the first phase of development (Table 1, upper grey box). Non-shaded features in Table 1 remain under consideration for future developments. Group members agreed that the website must be mobile and tablet device responsive, and that consent to future data use should be based on the GA4GH DUO. The fonts, colour schemes and layout were selected in accordance with accessibility principles and standards.

**Table 1:** Dynamic Consent platform features considered.

**Personal details and contact preferences**^a,b^
**Granular, Dynamic Consent choices**^a,b,c^
**Access to patient surveys**^a^
**Study updates and news**
**Two-way communication between participants and researchers**^a^
Consent guides^b,c^
Patient forum^a^
Educational materials^c^
Self-reported health information^a,b^
Representation of genomic testing results
Other research participation opportunities^b^
Tracking function for research samples and data

Features in bold texts were selected for inclusion.

^a^Ref. [20].

^b^https://www.peerplatform.org/.

^c^Ref. [22].

The findings of Curve’s independent work to gain an understanding of the main challenges with the conventional research consent process through the interview process is shown in Table 2. The recurring themes were that research consent forms are long, too wordy, and present complex information that patients do not read, and that consent relies on trust in the clinical provider rather than on understanding of the research project [23, 24]. Using standard methods of consent, patients do not get true choice, rather they are asked to consider a broad, ‘all in or all out’, one-off decision-making consent event. It was also highlighted that the consent process needs to be tailored to individuals, and that patients need to be given the right information at the right time, for example a refresher immediately before receiving their genomic testing results, on what kind of results they may receive, since it may be weeks or months since having given consent for testing.

**Table 2:** Stakeholder interview emerging theme areas: challenges to the consent process, and opportunities for Dynamic Consent.

Challenge	Opportunity for Dynamic Consent
Arranging appointments.	One interviewee estimated >90% patients prefer to arrange appointments via email, indicating study participants have a level of comfort with and access to technology.
The genetic counsellor and clinicians explain genomics by using their own phone to show videos.	Standardise the use of technology in health and digital resources.
It is important to tailor the consent discussion to the family, based on their motivations for having the testing and expectations.	De-prioritise sections of text that aren’t relevant to the patient, while providing a means to access the information at a later time.
If results are negative, it is left up to the patient to follow-up on what to do next.	Tailored and trusted information provided to participants that covers their whole research journey in one place.
Participants need to be able to make changes to their involvement in a study without contacting the genetic counsellor every time.	Easy access to the details of study participation and managing levels participation.
Keeping participants involved and motivated to do follow-up surveys.	Patient engagement through opportunities for ongoing interaction with the study. Digital reminders.
Consent forms are long and the language too difficult.	Online delivery formats can condense information. Online glossaries.
Participants need to get information at the right time, for example the different types of results explained just before receiving results.	Participants can return to review study information at any time.
Participants need time frames and an ability to check in with the study.	Two-way contact with researchers. Communication of study timelines, for example sample tracking.
Genetic reports are difficult to understand.	Online access to reports to promote discussion with health professionals.
Consent information is different between different studies. Participants do not read the consent form, they rely on the genetic counsellor to explain it.	Promote consistent presentation and language.
Tracking symptoms.	Patient reported outcomes.
Having access to support groups and information.	Providing participants with information and support from trusted sources.
Patients do not look at the consent form and probably do not understand the information. They base their decision to consent on trust in their doctor.	User friendly, interactive formats and organisation of information.
The ideal consent form would be 2–3 pages.	Organising study information in ways participants can focus on information that is priority for them.
Consent forms are difficult for non-English speakers. Downloading an app is another potential barrier.	Language translations and IT guides.

This analysis matched Australian Genomics’ experiences in delivering the genomic research program, and highlighted a number of areas where there was opportunity for the Dynamic Consent platform to address the identified challenges (Table 2). The findings also harmonised well with the working group’s selection of features to prioritise for the development of CTRL. For example, there was a strong imperative to limit the key information to short and simple statements, with each consent statement accompanied by an expandable ‘further information’ section for participants who felt they needed more detail. Information presented in this way cuts down the ‘walls’ of text that participants are faced with when working through paper-based consent forms, and the presentation format helps an individual to tailor the consent information to their own needs. The stepwise process moves away from the block of information with one final decision, instead breaking down important points into smaller decisions. Participants can absorb simple statements, checking them off as they go. In addition, with a simple login procedure, patients can return to the information at any time, including to refresh their memory on elements of the study. CTRL was developed to maintain an open communication channel with participants so they can continue to think about their choices and their research participation and understand what the results of the project are, which may influence decisions to participate in future research activities.

**Fig. 1:**
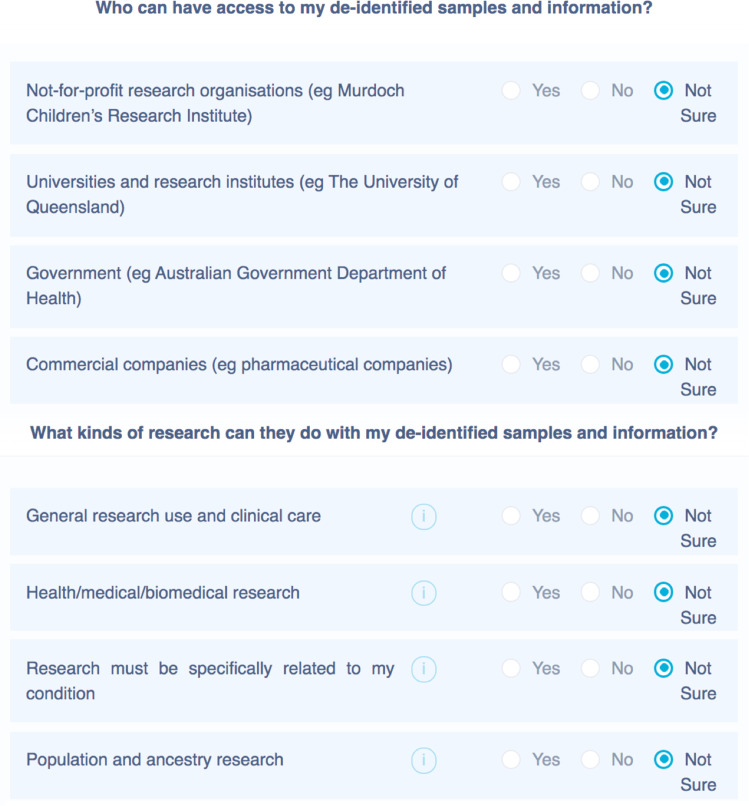
Future data (and biological sample) sharing preferences based on the Global Alliance for Genomics and Health Data Use Ontology (DUO) standard. Employing the DUO standard is expected to reduce ambiguity about the meaning of consent and facilitate inter-jurisdictional data sharing. The choices could have been designed so that for each research organisation participants may choose the kinds of research their data could be used for, but this was considered too complex, nor in keeping with the original planned use of DUO codes.

Delivery of Dynamic Consent questions was simplified beyond that originally planned. Rather than giving participants the opportunity to select what kinds of research projects each organisation could do using their data, which would result in a complex matrix of choices, we decided to more simply ask participants to select the organisations they approve and what types of research they approve (Fig. 1). More complex consent might be intimidating or too time-consuming, and the delivery approach chosen ultimately aligns more closely with the GA4GH DUO standard.

### Development

At the end of each development sprint, automated and manual user testing was carried out. Manual user testing involved volunteers and colleagues who, where possible, were not involved in the project, or did not have too much prior knowledge of it. Through this testing, a range of problems was reported at different times, both technical, and those related to user experience (Table 3). Reported issues were reviewed and fed back to the developer. Testers’ suggestions for solutions to encountered problems were also considered. Changes and suggestions that were considered to extend the scope for the project had to be evaluated in the context of project timelines, the fixed budget and overall applicability to the user group.

**Table 3:** Summary of problems identified using an automated and manual user testing protocol—after design sprints and final end-to-end testing.

Design
Colour schemes not bold enough Unclear text—too small Radio button designs confusing No indication of whether data are saved if hit back and next buttons Video accessibility function (subtitles) Video time stamp Meaning of ‘not sure’ unclear
Technical
Incompatibility with older browsers Broken links Links not opening new tabs or windows

The initial project spanned 12 months from commencement to launch of the first version of the website. The build phase took about 5 months. Completion of the build was followed by end-to-end user acceptability testing before deploying the website for use. CTRL was launched when it was agreed that the developer had delivered the scope of the project, and with the support of all stakeholders. Further planned developments and changes to address issues that have arisen later have led to ongoing deployment of new versions.

### The product—CTRL

On registration and first login, the user is guided through a five-step consent ‘wizard’. The first step is a 3-min animated video explaining Dynamic Consent and the reasons Australian Genomics is trialling the approach (also available on YouTube: youtube.com/watch?v=Du8kYb_9AKY). In the next four steps, participants are asked to confirm their understanding of, and make choices about: (1) having the clinical genomic test; (2) participating in the research; (3) preferences for return of results and (4) preferences for future sharing of their biological samples and data. In (3) and (4), participants do not have to provide an upfront ‘yes’ or ‘no’ answer, but can defer decision-making by selecting ‘not sure’. Choosing ‘not sure’ is equivalent to selecting ‘no’, and so prompting participants to revisit choices through follow-up by the study team may be employed as a future strategy to confirm participant choices. On completion of the consent wizard they are directed to the dashboard, which shows research participation tasks that have been completed and those tasks still to be completed, arranged in order of progression through the study. On all subsequent logins the landing page is the dashboard, from which the participant can access all pages relating to consent choices to update their preferences.

**Fig. 2:**
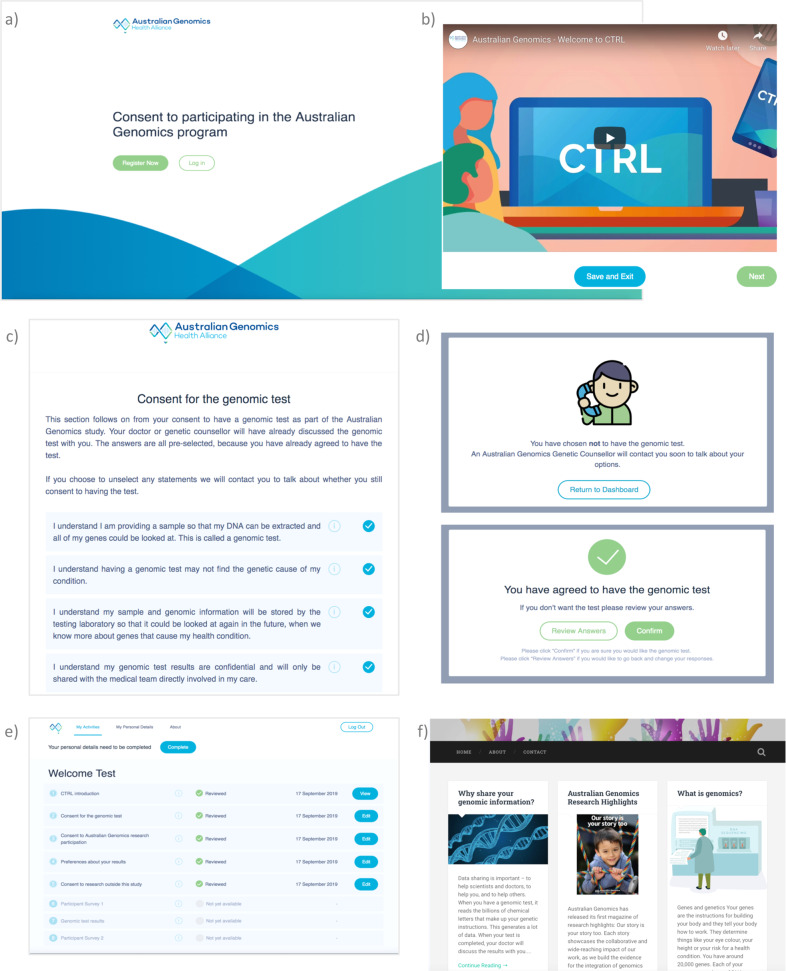
CTRL platform screenshots. Selected steps of the platform are shown **a** CTRL landing page, **b** animation describing Dynamic Consent, **c** consent to genomic testing, **d** choices are confirmed using pop up boxes, **e** the dashboard, **f** news and information. Demo the full platform at https://demo-ctrl.australiangenomics.org.au/ (contact authors for instructions).

A personal details page invites participants to enter their details and contact preferences. There is a page where participants can write messages to the research team (which then triggers two-way email contact), and keep up to date with news and information relevant to their involvement in the study. Regularly refreshed news and information is mirrored into the website through a separately maintained Wordpress blog. This method was chosen so that the study team could independently update news stories without the involvement of the developers to deploy new content. Screenshots from the CTRL platform are shown in Figs. 1 and 2 (or can be accessed at demo-ctrl.australian genomics.org.au—contact the author for instructions).

### HREC approvals

HREC approval was sought to allow participants in the Australian Genomics research program to start to use CTRL. An overview of CTRL was presented to the HREC at their Committee meeting. From the HREC point of view, website security and data privacy were key issues, as well as managing inequity of access for culturally and linguistically diverse participants or those with limited access to, or experience with, technology [25]. In our study a paper version of information and consent materials will always be made available, to address these concerns.

To reduce administrative burden and delay in the timing of new deployments of CTRL, it was specified in the research protocol that ethics approvals will only be sought where amendments to the platform would change the meaning or intent of the information or consent provided. Correcting typographical or grammatical errors, and new features or content without direct ethical implications (such as changes to the news and information section) will not be submitted for scientific and ethical review. This is in line with existing standards for ethical review where researchers send study updates to participants by mail or email, or have a participant-facing study website.

### Using CTRL in the Australian Genomics study

**Fig. 3:**
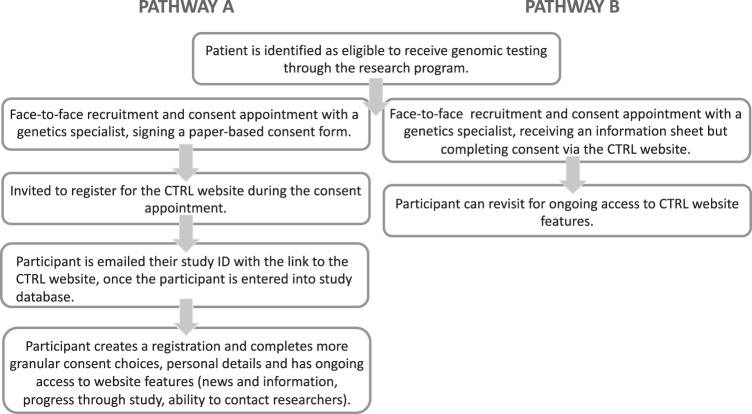
Incorporating Dynamic Consent into the existing Australian Genomics study participant pathway. Pathway A reflects the current implementation strategy. Pathway B is also HREC-approved, but barriers experienced include lack of access to tablets or computers in the clinic and inability to pre-assign participant Study ID numbers, which is not compatible with ethics requirements and standard study practice.

The model for implementation of the Dynamic Consent platform is not to use it as an independent tool initially, and not to fully replace paper-based consent, but to supplement it (Fig. 3). In the predominant pathway, participants are told about the opportunity to use CTRL during their face-to-face study recruitment appointment, and they create their registration by themselves afterwards. Therefore, CTRL in the current implementation is usually used as a post-consent and counselling tool for ongoing participant engagement and communication, as well as study and data management, rather than to support pre-counselling or in-person genetic counselling. Offering the use of CTRL in this way was partly done to allow us to test the system before implementing it fully, and partly to align with already approved protocol and consent materials, and the current ethical guidelines for consent forms. This delivery method also acknowledges that some participants may prefer not to use CTRL but instead will be satisfied with the consent they provided using paper-based forms. The self-directed nature of the use of CTRL by participants provides an excellent opportunity to evaluate participant preferences about using such tools.

The premise of a Dynamic Consent platform is that participants can make changes to their choices that will be reflected in real time. While an administrator interface for the portal is still under consideration as part of further development, three overlapping pathways have been established to allow the study team to access information in the CTRL website database and process changes in consent choices: (1) daily email reports generated by the platform that detail any new or changed registrations and changes to consent choices, (2) a securely hosted instance of Metabase, an open-source data analytic tool to view, extract and upload relevant data to the REDCap study database and (3) directly integrating crucial, time-sensitive information from the CTRL database into corresponding fields in the participant’s REDCap study database profile. This means REDCap can be queried at any time to determine participants’ consent preferences, and likewise access the complete history of changes made to consent choices. These methods reliably inform us of any actions that need to be taken by the study team, for example follow-up phone calls to participants.

## Discussion

### CTRL evaluation study

Australian Genomics developed CTRL for participants to use as part of their involvement in the study and to test whether it provides advantages over current consent models. CTRL is now open for participant registrations, with analytics and data on usage and choices made being gathered. Given that enrolment in CTRL is an optional step undertaken subsequent to participants providing broad consent for participation in future research, our evaluation will provide insights into ethical questions by providing data on how many participants want extra information, ongoing contact with the study and access to more granular decision-making. Changes to usage or choices over time may indicate if they find the ongoing contact or decision-making an over-burden. Participant choices about future research use of their information will indicate whether providing too much choice limits the availability of datasets for future scientific discovery. Patient experience will be evaluated through patient surveys (using validated measures of trust in research and researchers, satisfaction with research, decisional regret and knowledge of genomic testing) that reflect the recently proposed Dynamic Consent evaluation framework [15]. Together, this data will determine the impacts of increasing participant choices and providing a platform for engagement with participants involved in research. The results of the evaluation, which will be the subject of a follow-up publication, are expected to provide amongst the first evidence on implementation outcomes of the Dynamic Consent approach.

### CTRL addresses the Dynamic Consent framework

While CTRL has been released as a minimum viable product at this stage, there was a focus on meeting the core features of the Dynamic Consent framework. CTRL facilitates ongoing participant-led management of their involvement in research, by providing the opportunity for participants to choose from more granular consent options and change consent choices over time (including for future use of their data). Participants can indicate preferences for the kinds of results they would want returned; whether they receive alerts about further research their data is shared to, and their preferred methods of contact. CTRL allows participants to develop greater understanding of the research through the animation video and ongoing information delivery through the news and information pages. Participants can also easily establish two-way contact with the research team using the CTRL messaging system.

### Challenges to the use of CTRL

Initially, the proposed approach to using CTRL was for the study recruiter and participant to work through the online consent together during the study recruitment appointment. We predicted early on that this would be limited by a lack of access to IT in clinic, and hesitated in expecting participants to use their own smartphones for this purpose. We also predicted that gradual adoption of CTRL would be more acceptable to the range of stakeholders involved: HRECs, hospital recruitment sites, study recruiters and participants. These factors led to a decision to offer the use of CTRL as a supplementary second step to the usual consent process. This has challenged the user flow, and strategies to encourage participants to register for CTRL have necessarily been developed. This includes a flyer that is given with the consent form, which explains the benefits CTRL provides for participants. Also, after a participant is recruited into the study and the participant profile created in the REDCap study database, the database generates an automated email to the participant providing the information they need to register. These processes also help to minimise the amount of time spent during the recruitment and consent appointment on explaining CTRL in an already complex and lengthy discussion, which was indicated to us as a concern by study recruiters.

Once participants enrol in CTRL, future sharing of their genomic and other health information for further research, in accordance with their choices indicated in CTRL, has implications for data governance and management. It has long been established in research protocols that once data are shared it cannot be retrieved, and whether this fits with the principles of Dynamic Consent has not been widely discussed. For example, an individual’s data could theoretically be removed from an online database cohort, but what is the expectation on this once it has already been shared? While online databases where analyses can be performed without download of data are very conducive to Dynamic Consent, another factor to consider is the technical readiness of current databases for inclusion and exclusion of participant data according to their decisions about approved data users and uses. Further, data users may find that accessing and using information from unstable datasets may affect the validity of their findings, for example if seeking to strengthen evidence on the clinical impact of a variant based on the number of representations of that variant matched to phenotypes in a database. Another remaining question is whether cohort sizes assembled for sharing will still be meaningful if too many granular consent choices are applied. In addition, will our interpretation of, and application of, the DUO codes limit commercial or government-led research, in turn limiting scientific discovery?

The ability to use Dynamic Consent platforms to automate communication with research participants is an attractive feature, particularly for ongoing follow-up communication in the longer term. One unresolved challenge is that if follow-up communication is automated from the platform it is not possible to know whether the participant situation has changed or they have passed away. Linking consent platforms with electronic medical records is a technical, organisational, security and ethical challenge which must be addressed, to realise such platforms’ full potential.

Another implementation challenge for the nearer future relates to authentication. Guidelines around acceptable levels of authentication for electronic consent platforms are becoming available and future iterations of CTRL should use more robust authentication. This must be balanced with ease of access for registered participants [26, 27]. Another barrier has been that registration to CTRL requires a participant to have already had their Study ID number generated which, according to research guidelines, cannot be generated until the participant has already consented to be part of the study. Future applications of CTRL will include a review of whether the Study ID needs to be a requirement for registration to CTRL.

It is recognised that participant preferences and expectations may depend on cultural background, age, health conditions, or access to and degree of comfort in using technology. Further, how to best tailor Dynamic Consent to culturally and linguistically diverse communities and research participants with low literacy must be addressed [28]. Future planned work will include translation into other languages to ensure that the website is accessible to a diverse range of participants.

The Dynamic Consent model is thus still evolving and our work has identified just some of the remaining practical issues to be addressed by future research and implementation.

### Project strengths

Given the novel and multi-disciplinary nature of this project, one of the identified strengths of the project was the relatively short time frame in which CTRL was delivered. This has been attributed to factors including the approach to project management and use of project management tools by Curve and the Australian Genomics teams, the Curve Way^TM^, dedicated points of contact within each organisation (product manager and project lead) and project backing by a working group to facilitate decision-making. The project was delivered with a discrete budget, which meant many compromises and solutions had to be reached. Problem solving was equally contributed to both the developers and Australian Genomics.

In terms of risks to delivering CTRL within the Australian Genomics program, one of the consistently predicted outcomes of Dynamic Consent is ‘consent fatigue’, which may result from the burden of ongoing contact or requests for decision-making [29]. However, it is acknowledged that Dynamic Consent platforms can equally incorporate broad consent approaches [8, 30], and the implementation strategy in the Australian Genomics study offers both alternatives. There is likely to be a spectrum of preferences across research participants. Therefore, our protocol enables flexible delivery of consent and engagement strategies to participants who may want different levels of autonomy, information and support, or who have specific wishes regarding future use of their data. This is a great strength of the research programme’s consent strategy and could enhance research outcomes as well as fulfilling our ethical responsibility to participants taking part in genomic research.

### Adapting the platform to other research programmes

A main objective of the project was to develop a prototype platform that is flexible, adaptable and scalable, allowing it to be easily applied to other research projects. CTRL has been built upon for the online recruitment of up to 10,000 couples for the Mackenzie’s Mission Australian Reproductive Carrier Screening Project (mackenziesmission.org). A licensing agreement that outlines our expectations for sharing CTRL with other research programmes has been developed. The agreement asks the Licensee to acknowledge Australian Genomics in publications, share information on the number of users registered to their instance (as a measure of combined impact) and to share derivative works back to us. This way, a suite of tools or modules can be developed, which allows future research projects to customise the platform. Licensing and further developing the platform using this model will ensure its improvement and availability at low cost for other research projects.

## Conclusion

Dynamic Consent has the potential to address challenges of consent, increased requirements for engagement and management of secondary use of data in genomic studies, biobanks and other medical research. Australian Genomics has developed a Dynamic Consent product that is now being used in the Australian Genomics study. The outcomes of the evaluation being undertaken alongside its use will inform its appropriate application to other research programmes, where it can be further built upon, improved and refined. This publication, along with the evaluation of user experience which will follow shortly, will help other research groups considering implementation of Dynamic Consent to learn from our experiences. CTRL’s expanded use will provide much-needed data on the effects of Dynamic Consent on participants, researchers and organisations, and whether it should be mainstreamed into research use.
